# Effects of Exercise, Rehabilitation, and Nutritional Approaches on Body Composition and Bone Density in People with Multiple Sclerosis: A Systematic Review and Meta-Analysis

**DOI:** 10.3390/jfmk8030132

**Published:** 2023-09-08

**Authors:** Natascia Rinaldo, Alba Pasini, Sofia Straudi, Giovanni Piva, Anna Crepaldi, Andrea Baroni, Lorenzo Caruso, Fabio Manfredini, Nicola Lamberti

**Affiliations:** 1 Department of Neuroscience and Rehabilitation, University of Ferrara, 44124 Ferrara, Italy; natascia.rinaldo@unife.it (N.R.); alba.pasini@unife.it (A.P.); sofia.straudi@unife.it (S.S.); andrea.baroni@unife.it (A.B.); fabio.manfredini@unife.it (F.M.); 2 Doctoral Program in Environmental Sustainability and Wellbeing, Department of Humanities, University of Ferrara, 44121 Ferrara, Italy; giovanni.piva@unife.it; 3 Unit of Nephrology, University Hospital of Ferrara, 44124 Ferrara, Italy; anna.crepaldi@edu.unife.it; 4 Department of Nursing, Instituto Maimónides de Investigación Biomédica de Córdoba (IMIBIC), 14004 Córdoba, Spain; 5 Department of Environment and Prevention Sciences, University of Ferrara, 44121 Ferrara, Italy; lorenzo.caruso@unife.it

**Keywords:** multiple sclerosis, body composition, bone mineral density, nonpharmacological treatments, exercise and rehabilitation, nutritional interventions

## Abstract

People with multiple sclerosis (pwMS) are affected by a wide range of disabilities, including a decrease in bone mineral density (BMD) and a worsening of body composition (BC), which negatively impact their quality of life quality. This study aims to analyze the effects of nonpharmacological interventions—in particular, physical activity, nutritional approaches, and rehabilitation—on BC and BMD in pwMS. This systematic review and meta-analysis was performed following the updated version of the PRISMA guidelines. In July 2022, five databases (MEDLINE, Embase, The Cochrane Library, Google Scholar, Web of Science) and gray literature were screened. Relevant articles published between 1 January 1990 and 1 September 2022 in any language were included. Outcomes of interest were anthropometric, BC measures, and BMD. The RoB 2.0 tool was used to assess the risk of bias. After duplicates elimination, 1120 records were screened, and 36 studies were included. A total of 25 articles were focused on physical activity and rehabilitation, 10 on nutrition, and 1 on multimodal intervention. One-third of the studies were judged to be at high risk of bias. The meta-analysis showed a high degree of heterogeneity due to the high variability in disease severity and intervention duration, intensity, frequency, and type. In general, no intervention showed consistent positive effects on BC. However, the most promising interventions seemed to be high-intensity training and ketogenic diets. Only a few studies considered BMD, and the results are inconsistent. Nevertheless, more studies are needed in order to confirm these results.

## 1. Introduction

Multiple sclerosis (MS) is an immune-mediated demyelinating chronic inflammatory disease of the central nervous system that affected approximately 2.8 million people worldwide in 2020, and its global prevalence has increased by 14.7% since 2013. Among all World Health Organization (WHO) regions, Europe has the highest prevalence rate of MS, with approximately 133 per 100,000 people affected [1]. MS is twice as common in women than in men, and the age of onset is generally between the third and fifth decade of life [2].

MS is responsible for a wide range of disabilities with various clinical manifestations that depend on the location and severity of the lesions. They usually include typical but also nonspecific symptoms such as weakness or numbness of the limbs, blurry or double vision, dizziness, fatigue, and gait disturbance [3], which severely impact people’s perceived quality of life [4]. The pathogenesis of MS is complex, but several risk factors have been linked with MS insurgence and progression, including both genetic susceptibility and environmental exposures [5]. To date, there is no definitive therapy for MS, but current treatments often consist of multidisciplinary approaches, including medications, symptomatic treatments, rehabilitation, lifestyle modifications, and psychological support [2].

Several studies have reported a high prevalence of overweight and obesity among people with multiple sclerosis (pwMS), mainly due to low energy expenditure caused by limited physical activity (PA) and the use of high-dose steroids during acute relapses [6,7]. However, these results are not consistent across all the studies, as some of them reported no significant differences in the mean body mass index (BMI) between healthy populations and pwMS [8,9].

Obesity has been found to be related both to the insurgence and the deterioration of MS. Childhood and adolescent obesity is reported to increase MS susceptibility, especially among females [10,11,12,13], and some studies have reported that there seems to be a faster rate of MS-related disability progression in patients with obesity [14,15]. However, a recent review by Schreiner and Genes [6] reported that insufficient data on this topic have been published, as the major analysis was conducted during the COVID-19 pandemic and included mostly low-powered observational studies [6]. Other anthropometric parameters have been found to be risk factors for MS. Central obesity, evaluated through waist circumference and waist-to-hip ratio, seems to be associated with a worsening of the disability level, evaluated through the expanded disability scale status (EDSS) [8,16,17].

In addition to the controversial results regarding the direct association between high BMI and MS, excessive weight and fat percentage (%F) are well known to be associated with several comorbidities, such as increased insulin resistance, blood lipid issues, cardiovascular diseases, depression, and other consequences [18]. Moreover, the decreased level of PA and pharmacological treatment can lead to the deterioration of the body composition (BC) with an increase in fat mass and a decrease in lean body mass [7,19], with a direct implication in the health of pwMS [20].

Lack of PA, increased inflammation, and the use of medications enhancing bone resorption and inhibiting osteoblastic activity (e.g., corticosteroids, anticonvulsants, benzodiazepines, 25-hydroxyvitamin D) could also be related to the higher prevalence of lowered bone mineral density (BMD) and osteoporosis in MS patients, thus highlighting the importance of considering bone health when dealing with these patients [21,22,23,24,25]. Specifically, multiple cohort studies have shown that pwMS have a significantly lower BMD at the femoral neck and the lumbar spine than healthy controls [21,25,26]. However, the determinants of lowered BMD in MS are still unclear [21], but an interrelationship among many contributing factors suggests a link between the increased level of disability and inflammation. MS is closely linked to osteoporosis not only due to lower BMD but due to the higher risk of falling in MS patients than in the healthy population [23,27]; indeed, a 50% incidence of falling at least once in 3–6 months has been reported among pwMS [23]. Many fall-risk factors caused by MS have been examined, such as imbalance and instability, impaired mobility, and generally increased disability rate [23,25,28].

Considering all the reported issues, interventions are needed in order to improve weight status, BC, and BMD in pwMS. Different strategies, especially those based on physical activity or rehabilitation interventions and dietary (D) approaches, have been proposed to manage the disease over time, slowing the progression of MS and reducing the number of relapses [29,30,31]. However, only a few studies have considered the effects of these treatments on BC, BMI, and BMD [32,33,34,35].

Recent evidence reported a positive relationship between PA interventions and MS course. Among the other benefits (i.e., feasibility, well tolerability, mood, etc.), a general reduction in BMI and %F has been reported in pwMS following specific physical exercises, such as general PA [36], Pilates [37], and aerobic training [38]. Additionally, some studies focused on D interventions reported an improvement in BC [39,40]. Despite these promising results, the effects are often controversial [39,41,42], and the positive effects of nonpharmacological treatments on BC have not been proven. Moreover, studies that consider the effects of nonpharmacological interventions on BMD in pwMS are scarce [43,44,45,46].

The main objective of this systematic review is to collect and analyze all the studies that investigated the effects of any nonpharmacological intervention, including PA, rehabilitation, and nutritional approaches, on BMI, BC, and BMD in pwMS.

## 2. Materials and Methods

This systematic review was conducted in accordance with the updated version of the preferred reporting items for systematic reviews and meta-analysis (PRISMA) guidelines [47]. Articles were included if they reported data on the effects of any nonpharmacological interventions on BMD and body measures in pwMS. The included articles were full-text articles of any language published between 1 January 1990 and 1 September 2022. The study was registered in the “International Prospective Register of Systematic Reviews” (PROSPERO code CRD42022337939).

### 2.1. Selection Criteria

The PICO model was used to establish the selection criteria: (i) Population: pwMS. (ii) Interventions: any nonpharmacological intervention. Interventions will include but will not be limited to exercise training interventions, physical therapy, nutritional and physiological interventions. (iii) Comparator: Control group that received no treatment or a standard treatment, healthy controls, or no control group. (iv) Outcomes: BMD and anthropometric measures. Specifically, we considered BMI, waist circumference, waist-to-hip ratio, and BC parameters, such as %F, fat-free mass, and lean body mass. The exclusion criteria were as follows: people with different pathologies were considered together in the same study; the studies did not report data on the post-interventions; the studies had an observational study design.

### 2.2. Literature Search

The following electronic bibliographic databases were searched for eligible articles: MEDLINE, Embase, The Cochrane Library (Cochrane Database of Systematic Reviews, Cochrane Central Register of Controlled Trials (CENTRAL), Cochrane Methodology Register), Google Scholar, and Web of Science (science and social science citation index). Gray literature was screened through research from institutional repositories or online platforms. The search strategy included only terms relating to or describing the intervention, combined with Boolean operators. The complete search strategy used for MEDLINE is reported in Table 1. The search terms were adapted for use with other bibliographic databases.

Data were extracted by two independent blinded reviewers (N.R. and A.P.). In the first step, studies were selected by screening the titles and abstracts considering the inclusion and exclusion criteria. In the second phase, the full articles were reviewed by the same two authors for inclusion in the review. Disagreements will be resolved by a third reviewer (N.L.) for final consensus.

### 2.3. Data Collection

Two reviewers (N.R. and A.P.) independently extracted the data from a properly developed electronic spreadsheet. The following data were extracted: identification of the authors; year of publication; study design; characteristics of the participants of both the intervention and the control group (age and sex, severity and type of MS, sample size, inclusion and exclusion criteria, sampling process) details of the intervention (type, time, frequency, duration, and intensity) and control condition; all measures related to outcomes (BMI, waist circumference, waist-to-hip ratio, %F, fat free mass, lean body mass, BMD); information for the assessment of the risk of bias.

The Risk of Bias tool 2.0 was used by two independent reviewers (N.R. and A.P.) to assess the risk of bias and applicability of the studies [48]. This tool was used to assess the risk of bias in patient selection, blindness, and randomization, and a three-way classification (low, some concerns, high risk for 5 domains) was provided for each included study.

### 2.4. Statistical Analysis

Data from the included articles are reported as number and percentage for categorical variables, mean or median, with standard deviation or interquartile range or 95% confidence interval according to how data were reported in the manuscripts.

Data for the meta-analysis were extracted and pooled using a random-effects approach, with the application of standardized mean differences for continuous outcomes. Heterogeneity was assessed via both the chi-square test and the I-squared statistic, considering an I^2^ value greater than 50% to indicate substantial heterogeneity. Data analyses were performed with MedCalc Statistical Software version 20.218 (MedCalc Software Ltd., Ostend, Belgium) and Review Manager 5.4 version.

## 3. Results

**Figure 1:**
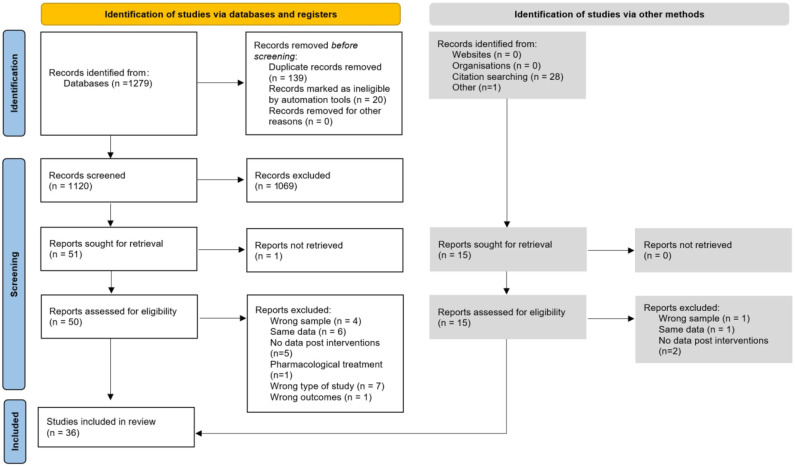
PRISMA flow diagram outlining literature review and study selection.

A total of 1279 records were identified, of which 159 were duplicates. After the screening of titles and abstracts, 1069 articles were excluded due to not meeting the inclusion criteria. The full texts of the remaining 51 papers were screened, and 24 articles were excluded. Then, 29 records were identified after citation searching and other resources, and 15 were assessed for eligibility. Of them, five were excluded. Finally, 36 articles were found to be eligible for inclusion. The study’s flow diagram is shown in Figure 1.

### 3.1. Characteristics of the Analyzed Studies

Table 2 presents the demographic and design details of the 36 included studies. According to the study design, 22 (61.1%) studies were randomized controlled trials (RCTs) [32,33,35,38,39,41,42,44,49,50,51,52,53,54,55,56,57,58,59,60,61,62,63,64,65,66], and 3 (8.3%) were non-RCTs [67,68,69]; 8 (22.2%) were pretest–posttest longitudinal trials [40,45,46,70,71,72,73,74] (of which 4 were single-group trials) [40,46,73,74]; and 3 (8.3%) were quasi-experimental open-label studies, among which 1 was defined as a “prospective, mixed and quasi-experimental study” [75] and 2 as “single-arm, uncontrolled, open-label pilot studies” [76,77]. Considering comparative studies among two or more groups (both RCTs and non-RCTs), 3 (8.3%) studies involved healthy people as comparator samples [67,68,69], while 14 (38.9%) involved other MS patients [32,33,35,38,39,49,50,51,53,55,56,60,61,62,63,65,66].

A total of 14 studies (38.9%) only considered MS patients with RRMS [32,38,39,40,42,50,51,59,63,67,69,71,72,73,77], while the other 12 papers (33.3%) also included different typologies of MS, such as SPMS [35,46,49,55,56,65,75,76,78], PPMS [33,55,60,65,75], CP [53,61,62], PRMS [46], or all MS typologies together [66]. Ten studies (28.6%) did not specify this detail [34,41,44,45,52,57,58,64,68,74]. In relation to the EDSS scale, 16 studies (44.4%) included patients with an EDSS score on average below 3.0 [32,39,40,42,51,53,57,59,62,63,64,66,67,68,70,72,74,77]. Conversely, nine papers (25.0%) did not specify these data in their studies [33,44,45,46,50,52,60,75,76], while one study (2.8%) only stated the selected range [69].

Most of the studies (19, 52.8%) considered 15 patients or fewer in the intervention group [33,38,41,44,45,50,52,53,57,58,60,62,65,66,67,69,70,71,76]. Only two studies (5.6%) involved more than 50 patients [40,72]. Almost all of the considered studies chose comparator groups with a sample size approximately equal to the intervention group. Nevertheless, two studies (5.6%) showed a substantial difference between samples, where the comparator group comprises half to one-third of the intervention group [61,63]. The female sex was the most represented among the considered studies. Indeed, the majority of them (97.1%) included over 50% of women in their intervention sample [32,33,34,35,38,40,41,42,45,46,49,50,51,52,53,55,56,57,58,59,60,61,62,63,64,65,66,67,69,70,71,72,73,74,75,76,77,78], and four studies (11.4%) only involved females [50,59,65,71]. No studies considered males and females separately. Twenty-two studies (61.1%) involved patients over 40 years of age on average [33,34,35,38,40,42,45,49,50,52,53,55,56,57,58,61,62,63,64,65,66,68,69,71,73,74,75,76,78], while two articles (5.6%) did not specify the mean age for their samples [44,60]. In addition to sex distribution, comparator groups mostly matched the intervention groups for mean age. Table 3 reports a summary of the studies’ characteristics.

### 3.2. Outcomes of Interest

The outcomes of interest considered in this study are BMI, BC parameters (which comprise waist circumference, waist-to-hip ratio, %F and fat free mass), and BMD. Of the 36 studies considered, 24 (66.7%) reported data on BMI pre- and post-intervention [17,32,38,39,40,41,42,50,55,58,59,60,61,64,66,67,68,69,73,74,75,76,81,82], and among them, 7 reported data only on BMI [39,42,55,58,64,67,76]; 26 studies reported data on %F (72.2%) [17,32,33,34,38,40,44,45,50,56,57,59,61,62,63,65,66,68,69,70,71,72,73,74,75,77,80], 8 reported data on waist circumference and/or waist-to-hip ratio (22.2%) [17,40,41,50,56,60,69,77], and only 4 (11.1%) analyzed BMD [34,44,45,46]. Among all the studies, 12 (33.3%) considered BMI or BC as the primary outcome [34,38,41,45,50,64,65,66,68,74,75,77], whereas all 4 papers that considered BMD analyzed it as the primary outcome [34,44,45,46]. Regarding the methods used for the evaluation of BMD, only the study of Yang et al. [46] used calcaneal quantitative ultrasound (QUS), whereas the other three studies evaluated it through dual-energy X-ray absorptiometry (DEXA) [34,44,45]. The methods utilized for the evaluation of %F were heterogeneous: DEXA in 12 studies (46.2%) [17,33,34,44,45,61,62,63,66,68,73,74]; bioelectrical impedance analysis (BIA) in 3 studies (11.5%) [32,38,57]; Bod-Pod in 2 studies (7.7%) [40,77]; and anthropometric method based on skinfolds thicknesses in 8 studies (30.8%) [50,56,57,59,65,72,75,80]. In 2 studies (7.7%), the authors did not report the methods used [69,81], whereas Dalgas et al. [57] used both BIA and skinfolds.

Concerning the mean BMI of the intervention groups, 8 fell into the normal-weight category (30.4%) [50,55,61,68,69,70,72,74], 13 into the overweight category (56.5%) [32,34,38,39,41,56,58,59,64,66,67,76,80], and 3 into the obese category (23.1%) [40,73,77]. Wens et al. [61] divided the sample into two intervention groups, one with a normal weight and one with an overweight mean BMI; whereas in their study, Riccio et al. [60] had three intervention groups, two normal weight and one overweight. In one study, the authors divided the sample between normal-weight and overweight subjects [42].

### 3.3. Types of Interventions

Regarding the PA interventions, the literature search identified four interventions based on a different kind of high-intensity training carried out for 8 or 12 weeks [38,61,66,68], two interventions based on Pilates for 12 [33] or 8 weeks [50], one hypertrophic training for 7 weeks [44], four interventions based on resistance and endurance training for 10 weeks [45] or 24 weeks [62,63,65], four on an aerobic training program for 8 weeks [59,67,69] and one for 12 months [72]. Two studies were based on lower extremity training, one for 8 weeks [82] and one for 12 weeks [57]. Two studies examined the effects of home-based PA, one high-intensity program [68], and one primarily walking [34], and both of them for 6 months. One study reported the effect of task-oriented circuit training for 14 weeks (two weeks supervised and 12 weeks at home) [55]. Finally, one study analyzed the effects of pragmatic physical interventions [41], and one was an individualized combination of stretching, aerobic, and endurance exercises [70]. Diet-based interventions were applied in 10 studies from the analyzed set (27.8%) [32,35,39,40,49,52,56,60,64,73,75,77,78]. Among these, six (60.0%) were randomized controlled trials [32,35,39,49,52,56,60,64,75,78]. Five trials (50.0%) administered a dietary protocol alone (e.g., ketogenic diet, hypocaloric diet) [40,52,64,73,75,77], while five studies (50.0%) opted for the administration of supplementation [32,39] or both [35,49,60,75,78]. For most studies (8, 80.0%), the trial duration was below 12 months [35,39,40,49,52,56,60,73,75,78]; among these, only one study presented an intervention shorter than 3 months [52]. The longest dietary trials were 12 months [64] and 24 months [32].

### 3.4. Effects of Physical Activity and Multimodal Intervention on BMI, BC, and BMD

Table 4 reports the characteristics and results of the PA interventions. The results revealed that the majority of high-intensity training programs had positive effects on BC with a reduction in %F and an increase in lean body mass [38,61,66]. The only exception is the high-intensity concurrent training by Keytsman et al. [74], which reported no changes in BC. Regarding the two studies that investigated the effects of Pilates, only one reported a significant reduction in BMI, waist circumference, and %F [50], whereas the study by Duff et al. reported no effects [33]. The effects of endurance and aerobic training are heterogeneous. Among the four studies that administered a resistance and endurance training program, only one showed a reduction in %F [65], two showed an increase in lean body mass [62,63] and one showed no changes in BC [45]. Aerobic interventions on pwMS were reported to decrease BMI and %F in all studies [59,67,72], except for the study by Castellano et al. [69], which reported no changes in BC, BMI, and waist circumference.

Six months of a high-intensity home-based program showed a reduction in weight and BMI in pwMS but no effects on %F and fat-free mass in both pwMS and healthy controls [74]; whereas internet-delivered PA (primarily walking) had positive effects on BMD and %F but no increase in lean body mass [34]. No effects on BMI have been reported for task-oriented circuit training [55]. Leisure exercise intervention, pragmatic physical intervention, and individualized combined PA intervention showed no effects on BMI [41,58], waist-to-hip ratio [41], and BC [70]; no effects on BC and BMI have also been found after 12 weeks [57] and 8 weeks [80] of lower-extremity resistance training.

One study tested the effects of a multimodal intervention on nine females with MS, combining 12 months of a modified Paleolithic diet with physical exercise, and reported a reduction in weight and BMI. However, no control group was analyzed [76].

Only four studies considered the effects of physical interventions on BMD [34,44,45,46]. Montealegre et al. [44] examined the effects of 7 weeks of hypertrophic training, reporting a reduction in BMD. A positive effect on BMD has been shown after 7 weeks of whole-body vibration training [46] and after 6 months of internet-delivered PA [34]. No effect on BMD has been reported after 10 weeks of resistance training [45].

**Figure 2:**
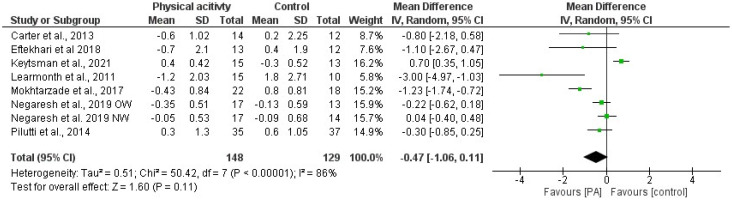
Forest plot of the meta-analysis on the effects of physical activity interventions on body mass index (BMI) in pwMS. Green dots represent the Mean Differences; the black lines represent the 95% CI. Included articles: Carter et al. [41]; Eftekhari and Etemadifar [50]; Keytsman et al. [66]; Learmonth et al. [58]; Mokhtazarde et al. [59]; Negaresh et al. [42]; Pilutti et al. [34].

**Figure 3:**
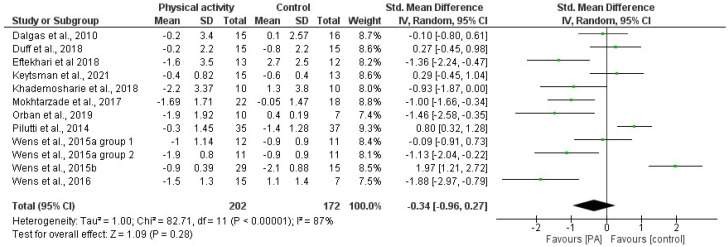
Forest plot of the meta-analysis on the effects of physical activity interventions on %F in pwMS. Green dots represent the Mean Differences; the black lines represent the 95% CI. Included articles: Dalgas et al. [57]; Duff et al. [33]; Keytsman et al. [66]; Khademoshaire et al. [65]; Mokhtazarde et al. [59]; Orban et al. [38]; Pilutti et al. [34]; Wens et al. [61,62,63].

Based on the results derived from the meta-analysis that analyzed the effects of PA interventions on BMI and %F, the standardized mean difference between the intervention groups before and after the intervention was estimated to be −0.37 (−0.80 and 0.06 CI) and −0.34 (−0.96 and 0.27 CI), respectively. The results did not indicate significant positive effects of PA interventions on BMI and indicated no effects on BC. In both cases, the heterogeneity was high (I^2^ = 84% for BMI and I^2^ = 87% for %F). Figure 2 and Figure 3 present the forest plots with the standardized mean difference index and its 95% confidence interval in each study, as well as the final estimation of the index from the combination of studies.

### 3.5. Effects of Diet/Supplement-Based Interventions on BC and BMD

Table 5 presents the characteristics and the results of the D interventions. Diverse effects of D interventions on BC and BMD have been registered. Two studies (20.0%) analyzed BMI as the only anthropometric outcome [64]. Similarly, two studies (20.0%) considered BC only [32,75], while five studies (50.0%) considered both [35,40,49,52,56,73,77,78]. Moreover, five studies (50.0%) also included circumferences among their anthropometric outcomes [32,40,52,60,77].

Among studies that analyzed BMI, a significant reduction in BMI consequent to interventions was registered in five cases (71.4%) [40,64,73,77]. Of these, three studies (60.0%) reported BMI reductions in both the intervention and comparator groups [35,39,49,56,64,78], although one of these reported a more significant and faster reduction in the control group [64]. Conversely, two studies (28.6%) did not report any changes in BMI from baseline or between groups [52,60]. Body fat was reduced in six of the considered studies (60.0%) [35,40,49,56,73,75,77,78], and in only two cases were no significant changes registered [32,52]. Interestingly, in one case [35,49,56,78], a decrease in %F was registered only for the intervention group, while BMI significantly decreased both in the intervention and control groups from baseline. Only in two cases (20.0%) was there a significant reduction in bodily circumference reported [40,77]. No studies highlighted a worsening of BC parameters over time, although two studies (20.0%) also reported a decrease in fat-free mass along with BMI, %F, and waist circumference [40,73,75,77].

Studies characterized by a diet-based trial without supplementations mostly administered a ketogenic diet [40,75,77]. All these studies registered a consistent reduction in all BC parameters (BMI, %F, fat free mass, waist circumference), as well as one of the other dietary interventions [73].

Among trials that administered dietary supplementations [32,39], only in one study was there a significant decrease in BMI, but it was registered for both the intervention and control groups [39].

Trials administering both diet and supplements registered, in most cases, a reduction in body fat after 4 months from baseline [35,49,56,75,78]. These studies specifically provided supplemental formulae based on polyphenol administration to increase ketone bodies. Only one of these mixed-dietary trials did not show any changes among groups over time [60].

**Figure 4:**
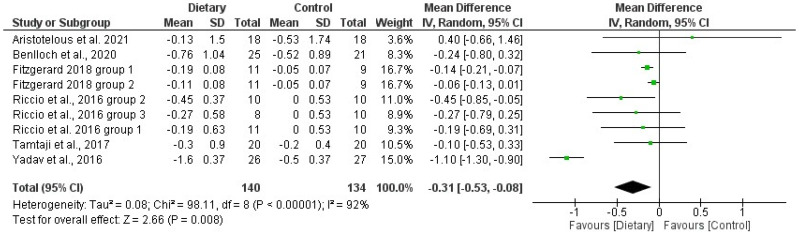
Forest plot of the meta-analysis on the effects of dietary interventions on body mass index (BMI) in pwMS. Green dots represent the Mean Differences; the black lines represent the 95% CI. Included articles: Aristotelus et al. [32]; Benlloch et al. [56]; Fitzgerard et al. [52]; Riccio et al. [60]; Tamtaji et al. [39]; Yadav et al. [64].

**Figure 5:**
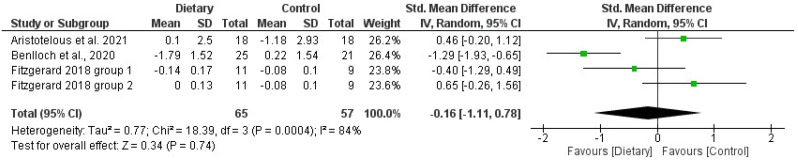
Forest plot of the meta-analysis on the effects of dietary interventions on fat percentage in pwMS. Green dots represent the Mean Differences; the black lines represent the 95% CI. Included articles: Aristotelus et al. [32]; Benlloch et al. [56]; Fitzgerard et al. [52].

The forest plots reporting the results of the meta-analysis that analyzed the effects of D interventions on BMI and %F are presented in Figure 4 and Figure 5. The standardized mean differences in the intervention groups before and after the intervention were estimated to be −0.31 (−0.53 and −0.08 CI) for BMI and −0.16 (−1.11 and 0.68 CI) for %F. The meta-analysis resulted in a significant positive effect of D interventions on BMI but no effect on %F. In both cases, the heterogeneity was high (I^2^ = 92% for BMI and I^2^ = 82%).

Concerning bone health, no studies administering dietary trials focused on changes in BMD.

### 3.6. Risk of Bias

Figure S1 of the Supplementary Material shows the risk of bias for each domain in all included studies assessed through the Rob tool. Only seven papers had an overall low risk of bias [32,33,55,57,61,63,64]. The majority of the studies (47.2%) overall were judged as having some concerns of bias, whereas 12 (33.3%) were judged to be at high risk of bias. The papers judged at high risk of bias were judged as such mainly because of the measures of outcomes and selections of the results; only two of them [60,76] had high risk in the randomization process and deviation from the intended interventions domains.

## 4. Discussion

This systematic review aims to investigate the state of research on the effects of any nonpharmacological interventions on anthropometric measures, in particular BMI and BC and BMD in pwMS. Only a few studies have examined the effects of nonpharmacological interventions on BC or BMD as either primary or secondary outcomes. The results of this review are based on 36 interventional studies, of which 22 are RCTs, 3 trials involved healthy control subjects, and 11 did not include a comparator group. The main nonpharmacological interventions are PA, rehabilitation or exercise interventions, dietary interventions, including probiotic supplementation, and one case of multimodal intervention [76]. Among them, the most common PA and exercise interventions are aerobic training, high-intensity training, Pilates, vibration training, and home-based exercises; whereas the majority of D interventions are supplementations (i.e., omega-3 and coconut oil and probiotic supplements) and diets (i.e., ketogenic, plant-based, and isocaloric).

The relationship between weight status and MS is complex and multifaceted. Some studies have reported a close relationship between childhood and adolescent obesity and MS susceptibility [6,10,12], especially in women [13]. However, even if the pathophysiological mechanism underlying this association has not been proven [81], several hypotheses have been proposed, mainly linked to the role of adipokines as possible modulators of the immune response [8] and the low level of vitamin D in children with high body mass. On the other hand, the association between high BMI and MS clinical outcomes is not well understood. A recent paper by Lutfullin et al. [14], which analyzed 1066 individuals with newly diagnosed MS from the German National MS cohort, reported that obesity was associated with higher disability at baseline and in the follow-up. Other studies confirm this association [15]. Moreover, obesity is associated with several comorbidities, such as coronary heart disease, noninsulin-dependent diabetes mellitus, lipid abnormalities, and bone loss [7]. PwMS have also been reported not to participate in adequate daily recreational or structured PA for several reasons, such as those arising from physical, economic, and emotional barriers [82], thus increasing their risk of weight-associated disorders [29]. In light of the importance of BMI as a proxy for weight and health status, future research should consider the BC of patients with MS, especially %F and fat free mass [18,43]. Even if recent studies reported no significantly higher BMI in pwMS than in healthy subjects [9,83], Wingo et al. [84] found that men with MS had a higher %F and less fat free mass than BMI-matched individuals.

Despite the importance of BC and BMD for the health and quality of life of pwMS, studies focused on these variables are scarce [7], and only 23 articles in this review considered BMI (10, 27.8%), BC (9, 25.0%), and BMD (4, 11.4%) as first outcomes. The majority of the studies in the scientific literature are focused on MS-related clinical outcomes, such as cognition impairment [85], fatigue and depression [79], and disability [86] for PA and on health clinical outcomes and fatigue and quality of life [87,88] for D and supplementation interventions [89,90,91,92,93].

Considering the studies analyzed in this review, not all of them reported a beneficial effect of nonpharmacological interventions on BC, with some finding no effects at all [32,33,39,41,42,45,51,52,55,57,58,60,69,70,74,80], or the effects are sometimes found in both the control and the intervention groups [53,62,64]; therefore, it can be associated with the specific intervention. Regarding PA interventions, the best results on BC are found in practice for 8 weeks of clinical mat Pilates [50] and high-intensity training for 8 or 12 weeks [38,61]. Additionally, 12 months of individualized aerobic endurance exercise has been shown to induce a decrease in %F, but the study lacked a control group, and the risk of bias was moderate [72]. No consistent results can be summarized for the other types of interventions. Regarding D interventions, one RCT study [64] showed the best effects on BMI through a 12-month administration of a low-fat plant-based diet, while the most significant improvement in BC was registered in another study [56] through the administration of a ketogenic/Mediterranean dietary plan over 4 months. Comprehensively, ketogenic dietary plans showed good results on BC and weight status [40,56,77]. Conversely, dietary interventions based on polyunsaturated fatty acid administration [32,39,60] did not show any change from baseline even after 24 months [32]. Moreover, only two dietary studies showed a low risk of bias [32,64]. In general, it is not objectively clear if a specific intervention is superior to others or whether a certain type, frequency, or duration is better in terms of positively influencing BC. Bisht et al. [76] are the only study that reported the effect of a multimodal intervention on the weight and BMI of pwMS. A combination of a modified paleolithic diet with supplements, stretching, strengthening exercise with electrical stimulation, meditation, and massage has been shown to significantly decrease the weight and BMI of patients. However, the sample size was very low, with no control group and a high risk of bias; therefore, more studies are needed to confirm these results.

Our meta-analysis indicated that no significant effect of PA on %F can be detected, although there was a tendency toward an improvement in BMI. D interventions showed similar results, with a significant positive influence on BMI but no significant effect on %F. It is important to consider the high heterogeneity between the considered RCTs, with I^2^ values of 84% and 87% for PA interventions and 92% and 84% for D interventions. This high heterogeneity in the results can be due to several factors. First, the different types of PA and D interventions, the different durations, the small sample size for the majority of the studies (fewer than 15 patients in 54.3% of cases) and that in pwMS training intensity analyses are limited by work capacity; therefore, the RCTs are not consistent for all patients. Another important confounding factor is that most of the studies had a sample comprising mostly females. This mirrors the higher frequency of MS among women than among men [94,95]. Indeed, an average sex ratio of 2.3–3.5-1 between women and men is reported for MS [96]. However, this could have biased the results, as it is well known that the effects of PA and D interventions on BC have different effects on the two sexes [97,98,99,100,101,102]. Therefore, future research should consider analyzing the two sexes separately. Moreover, it is important to consider the high variability in multiple sclerosis-related drug intake, which could influence both the BC and the BMD of the patients.

Although, to our knowledge, this is the first systematic review to analyze the effects of nonpharmacological treatments on BMI, BC, and BMD in pwMS, other reviews partially analyze the effects of PA on BC in pwMS and find similar results. Mokhtarzarde et al. [18], analyzing a limited number of papers, concluded that the scientific literature did not support the positive effects of PA in pwMS. A recent review that analyzed the effects of Pilates in patients with MS underlined its role in improving BC, muscle strength, and core stability [37]. Ewanchuk and colleagues conducted a scoping review investigating the effects of PA on vascular comorbidities in pwMS. The results focused on BC reported substantial variability in the outcomes; in particular, the PA interventions seemed to be ineffective on BMI but could decrease %F if the intervention duration was at least 12 weeks [36]. Regarding D interventions, Mische et al. concluded that the Mediterranean diet, due to its strong relationship with cardiovascular comorbidities, should be employed in pwMS, but other studies are needed to confirm this hypothesis [91].

Regarding the effects of nonpharmacological interventions on BMD, studies are scarce and often inconclusive. Low BMD can be considered a primary cause of concern in MS due to the higher prevalence of hospitalization, impaired quality of life, and mortality for MS people compared to populations without this pathology [22,23,103,104]. All these elements together undoubtedly contribute to considering osteoporosis prevention a major point of interest, especially when considering that up to one-third of pwMS have been diagnosed with osteoporosis [25]. Given their increased risk for falling and lowered BMD, MS patients are exposed to a high risk for bone fractures [105,106]. PwMS indeed experience at least one fall over 12 months in 60% of cases, usually as a direct consequence of low-impact traumas caused by falls [24]. Fracture onset in pwMS has been investigated through several cohort studies, which stated an incidence between 1.43% and 6.2% [24]. A recent, wide cohort study involving over 1200 subjects [107] underlined how osteoporosis fractures are more common in pwMS than in other patients (47.4% vs. 34.2%). Despite the undoubted concern about low BMD in pwMS, we found only four studies that analyzed the effects of PA on BMD [34,44,45,46] and no studies on D interventions. Only one of the four studies was an RCT [34]; therefore, no meta-analysis could be performed. Pilutti et al. [34] reported that 6 months of internet-delivered PA increased BMD in comparison to pwMS who did not have any behavioral intervention. Additionally, 8 weeks of whole-body vibration training seemed to increase BMD [46]. On the other hand, resistance training and hypertrophic training did not have any effect or decreased BMD in MS patients [44,45]. It must be considered that the risk of bias was high in all three non-RCTs and moderate in the RCT; therefore, other RCTs need to be conducted to determine the effects of PA on BMD. Considering the promising results obtained through exercise on BMD in multiple studies in healthy and MS subjects [108,109,110,111], a further investigation concerning pwMS on this topic should be carried out. Indeed, it is well known that exercise positively influences bone metabolism [112]. However, given the multi-etiological susceptibility of pwMS to osteopenia and osteoporosis, the need for specifically tailored protocols should be considered.

This review has some limitations. First, the studies often reported different outcomes; therefore, we limited our main analysis only to %F and BMI; patients’ BC and BMD were evaluated through different methodologies, which could have introduced some bias. Second, all the meta-analyses reported had a high degree of heterogeneity. This aspect, despite limiting the overall generalizability of the findings, was not solvable even after the analyses of the funnel plots and after taking into account Cochrane’s recommendations (Schroll et al., 2011; [113]). The source of heterogeneity is probably derived from the high variability of disease severity and phenotype and of interventions’ duration, intensity, frequency, and type. In addition, the exercise intensity was simply reported as defined by the authors of each manuscript. In any case, it needs to be noted that high-intensity exercises were adapted for pwMS, taking into account the facts that pwMS fatigue quite easily and may lose motor control during training execution.

Finally, some of the studies had no control group or used healthy subjects as a comparator; therefore, they could not be included in the meta-analysis.

## 5. Conclusions

In conclusion, this is the first systematic review that investigates the effects of any nonpharmacological treatment on BMI, BC, and BMD in pwMS. The majority of the collected studies included PA and D interventions, and only one analyzed the effects of a multimodal intervention that combined diet, physical exercise, and rehabilitation. Due to the high heterogeneity of the studies and the overall low quality of the evidence, it is difficult to summarize the results, and in general, neither PA nor nutritional interventions showed consistent positive effects on BMI and BC in patients with MS. However, high-intensity training and ketogenic diets seemed to have promising effects on the improvement of BC. Regarding BMD, the results are even less consistent, and only a few studies have taken into consideration this outcome, despite its importance in pwMS. In conclusion, more RCTs examining the effects of nonpharmacological treatments are recommended in order to understand the potential of these interventions in improving body measures and decreasing the risk of low bone health and osteoporosis in pwMS.

## Figures and Tables

**Table 1:** Search strategy for MEDLINE.

Component 1		Component 2
“Multiple Sclerosis” [Mesh] OR “Multiple Sclerosis” [Mesh:NoExp] OR “multiple scleros*” [tiab] OR “Multiple Sclerosis, Relapsing-Remitting” [Mesh] OR “PwMS”	AND	“Body Weights and Measures” [Mesh] OR “Body mass index” [Mesh] OR “Body Weights and Measures” [Mesh:NoExp] OR “Body mass index” [Mesh:NoExp] OR “Body Composition” [Mesh] OR adiposity [tiab] OR “body fat” [tiab] OR “Waist Circumference*” [tiab] OR “Skinfold* Thick*” [tiab] OR “body compos*” [tw] OR “Bone Density” [Mesh] OR “mineral density” [tiab] OR “bone adj1 density

**Table 2:** Demographic details from the considered studies.

	Study	Study Design	Intervention Group				Comparator				Inter. Type	Outcomes of Interest
			Total Sample - %Females	Age	MS Type - EDSS	BMI, BC and BMD At Baseline	Total Sample - %Females	Age	Pathology - EDSS	BMI, BC and BMD At Baseline		
1	Straudi et al., 2022 [55]	RCT	18 - 38.9	49.7 ± 13.6	27.8% PPMS 27.8% SPMS 44.4% RRMS - 4.6 ± 0.7	BMI: 24.3 ± 4.13	18 - 33.3	52.6 ± 12.6	33.3% PPMS 27.8% SPMS 38.9% RRMS - 4.8 ± 0.6	BMI: 26.3 ± 4.0	P	BMI
2	Brenton et al., 2022 [40]	Single-group pre-test–post-test trial	65 - 84.6	40 (15–54) °	RRMS - 2.3 ± 0.9	BMI: 33.2 ± 7.0 FM: 41.3 ± 16.1 FFM 51.9 ± 10.7 WC 104.8 ± 14.2	-	-	-	-	D	BMI, WC, %F (Bodpod)
3	Aristotelous et al., 2021 [32]	RCT	18 - 50.0	39.1 ± 8.7	RRMS - 2.2 ± 1.1	BMI: 25.1 ± 4.4 %F: 29.4 ± 7.0	18 - 60.0	38.1 ± 5.3	RRMS - 2.36 ± 1.09	BMI: 25.1 ± 5.4 %F: 29.0 ± 9.2	D	BMI, BC (BIA)
4	Keytsman et al., 2021 [66]	RCT	15 - 60.0	41 ± 9	Any - 2.0 ± 1.3	BMI: 25.1 ± 3.2 %F: 30.3 ± 8.8 FFM: 48.2 ± 8.2	13 - 61.5	43 ± 9	MS - 2.7 ± 1.3	BMI: 27.4 ± 3.4 %F: 35.9 ± 4.9	PA	BMI, %F (DEXA)
5	de la Rubia Ortí et al., 2021; [49] Platero et al., 2021 [78], 2020 [79]; Benlloch et al., 2020 [56]	RCT	25 - 81.5	44.6 ± 11.3	74.1% RRMS 25.9% SPMS - 3.37 ± 2.03	BMI: 25.9 ± 5.3 %F: 19.3 ± 4.0 WHR: 0.89 ± 0.10 WHTR: 0.57 ± 0.08	21 - 58.3	49.83 ± 12.42	70.8% RRMS 29.2% SPMS - 3.8 ± 2.00	BMI: 25.7 ± 6.0 %F: 19.1 ± 5.0 WHR: 0.95 ± 0.08; WHTR: 0.60 ± 0.08	D	BMI, %F (skinfolds, diameters, perimeters), WHR, WHTR
6	Montealegre et al., 2020 [44]	RCT	5	-	SPMS	BMD: 2.03 ± 0.39 g/cm^2^ FM: 29.45 ± 8.35	-	-	-	-	PA	BC, BMD (DEXA)
7	Wingo et al., 2020 [73]	Single-group pre-test–post-test trial	20 - 85.0	46.2 ± 11.6	RRMS - 3.3 (2.0, 4.4) °	BMI: 34.7 ± 6.4 FM:43.2 ± 11.0 LBM: 48.2 ± 10.2 WC: 110.4 ± 13.9	-	-	-	-	D	BMI, %F (DEXA)
8	Benlloch et al., 2019 [75]	A prospective, mixed and quasi-experimental pilot study	27 - 81.5	44.6 ± 11.3	74.1% RRMS; 22.2% SPMS; 3.7% PPMS - N.A.	%F: 19.5 ± 3.8	-	-	-	-	D	BMI, %F (skinfolds)
9	Brenton et al., 2019 [77]	Single-arm, uncontrolled, open-label pilot study	20 - 85.0	38 (15–50) °	RRMS - 2.2 ± 0.9	BMI: 34.1 ± 6.9 FM: 42.5 ± 16.6 FFM: 51.1 ± 10.8	-	-	-	-	D	BMI, WC, %F (Bodpod)
10	Keytsman et al., 2019 [68]	Non-randomized trial	18 - 33.3	41.7 ± 8.5	N.A. - 1.9 ± 1.1	BMI: 24.8 ± 3.9 %F: 23.8 ± 9.6 FFM: 51.1 ± 7.4	19 - 26.3	41.5 ± 9.9	Healthy	BMI: 24.6 ± 2.8 %F: 22.7 ± 7.5 FFM: 53.3 ± 9.8	PA	BMI, %F (via DEXA)
11	Keytsman et al., 2019 [74]	Single group pre-test–post-test trial	16 - 56.2	52.8 ± 7.2	N.A. - 2.6 ± 1.5	BMI: 23.5 ± 3.3 %F: 29 ± 6.7 LBM: 44.2 ± 10.7	-	-	-	-	PA	BMI, %F (via DEXA)
12	Orban et al., 2019 [38]	RCT	10 - 90.0	44.7 ± 9.4	RRMS - 3.5 (2.5–4)	BMI: 26.9 ± 4.4; %F: 32.9 ± 11.9; FFM: 46.6 ± 7.0	7 - 75.0	48.7 ± 8.4	RRMS - 3 (2–4)	BMI: 29.6 ± 7.1; %F: 23.3 ± 7.1; FFM: 61.8 ± 9.7	PA	BMI, %F, lean body mass (BIA)
13	Pareja et al., 2019 [45]	Pre-test–post-test longitudinal design	11 - 63.6	46.5 ± 6.9	-	F%: 37.1 ± 7.1 FFM: 40.3 ± 7.6 BMD: 1.09 ± 0.15 g/cm^2^	-	-	-	-	PA	BMD, %F (DEXA)
14	Barry et al., 2018 [67]	Non-randomized trial	9 - 88.9	35.3 ± 2.1	RRMS - 2.2 ± 0.40	BMI: 27.9 ± 2.1	10 - 80.0	36.0 ± 2.0	Healthy	BMI: 24.6 ± 1.2	PA	BMI
15	Duff et al., 2018 [33]	RCT	15 - 80.0	45.7 ± 9.4	93.3% RRMS, 6.7% PPMS - N.A.	%F: 32.7 ± 8.3; FFM: 50.9 ± 11.6	15 - 73.3	45.1 ± 7.4	73.3% RRMS, 13.3% SPMS, 13.3% PPMS - N.A.	%F: 32.2 ± 10.5; FFM: 51.7 ± 11.5	PA	BC (DEXA)
16	Eftekhari and Etemadifar, 2018 [50]	RCT	13 - 100.0	34.5 ± 7.3	RRMS - N.A.	BMI: 24.4 ± 5.4; %F: 35.1 ± 9.5; FFM: 37.3 ± 3.7; WC: 85.2 ± 16.9; WHR: 0.83 ± 0.08	12 - 100.0	31.41 ± 8.89	RRMS - N.A.	BMI: 24.66 ± 4.64; %F: 36.2 ± 6.1; FFM (kg): 40.1 ±4.8; WC: 87.4 ± 11.5; WHR: 0.85 ± 0.07	PA	BMI, WC, HC, WHR, BC (skinfolds)
17	Khademosharie et al., 2018 [65]	RCT	10 - 100.0	20–50	PPMS, SPMS - 3.1 ± 0.5	Weight: 60.8 ± 13.3; %F: 36.3 ± 8.6	10 - 100.0	20–50	PPMS, SPMS - 3.8 ± 1.1	Weight: 59.7 ± 11; %F: 33.6 ± 8.1	PA	Weight, %F (skinfolds)
18	Negaresh et al., 2019 [42]; Mokhtarzade et al., 2018 [51]	RCT	Group 1: 17 - 64.7	31.2 ± 3.1	RRMS - 1.5 ± 0.8	BMI: 21.4 ± 0.8	Group 1: 14 - 64.3	29.1 ± 3.0	RRMS - 1.4 ± 1.0	BMI: 21.8 ± 1.6	PA	BMI
			Group 2: 17 - 64.7	32.1 ± 2.1	RRMS - 1.8 ± 0.8	BMI: 27.7 ± 1.3	Group 2: 13 - 69.2	32.2 ± 3.3	RRMS - 1.7 ± 1.2	BMI: 28.3 ± 1.3		
19	Fitzgerald et al., 2018 [52]	RCT	Group 1: 11 - 81.8	Group 1: 40.5 ± 5.4	-	%F: 47.4 ± 7.8; FFM: 45.0 ± 11.2; WC: 104.3 ± 21.4;	9 - 66.7	33.3 ± 7.0	MS (Any type)	%F: 44.9 ± 4.6; FFM: 45.2 ± 9.7; WC: 101.1 ± 16.6	D	BMI, BC (DEXA), WC, HC
			Group 2: 11 - 81.8	Group 2: 38.5 ± 7.4	-	%F: 44.8 ± 7.3; FFM: 44.3 ± 7.8; WC: 96.4 ± 10.9						
20	Yang et al., 2018 [46]	Single-group pre-test–post-test longitudinal design	22 - 72.0	50.3 ± 14.1	16% RRMS; 5% SPMS; 1% PPMS; 3% UNMS - N.A.	BMD T-score: 0.61 ± 1.85 (stronger side)	-	-	-	.	PA	BMD (QUS)
21	Mokhtarzade et al., 2017 [59]	RCT	22 - 100.0	32.0 ± 2.81	RRMS - 1.84 ± 0.35	BMI: 27.1 ± 2.5; %F: 34.66 ± 5.68	18 - 100.0	31.27 ± 3.28	RRMS - 1.57 ± 0.64	BMI: 26.2 ± 1.7; %F: 35.4 ± 4.5	PA	BMI, %F (skinfolds)
22	Tamtaji et al., 2017 [39]	RCT	20 - N.A.	32.8 ± 9.2	RRMS - ≤4.5	BMI: 25.6 ± 4.6	20 - N.A.	34.9 ± 8.9	RRMS - ≤4.5	BMI: 24.7 ± 3.7	PS	Weight, height, BMI
23	Wens et al., 2017 [53]; 2015 [61]	RCT	Group 1: 12 - 58.3	43 ± 3	RRMS: 18; CP: 5 - 2.3 ± 0.3	Group 1: BMI: 26.1 ± 1.14; %F: 36.2 ± 1.9; FFM: 48.5 ± 3.1	11 - 81.8	47 ± 3	RRMS: 8; CP: 3 - 2.5 ± 0.3	BMI: 27.0 ± 1.4; %F: 38.2 ± 2.1; FFM: 43.2 ± 2.1	PA	BC (DEXA),
			Group 2: 11 - 54.5	47 ± 3	RRMS: 18; CP: 5 - 2.7 ± 0.3	BMI: 24.4 ± 1.2; %F: 33.6 ± 2.8 FFM: 45.4 ± 2.6						
24	Riccio et al., 2016 [60]	RCT	Group 1: 11 - 90.9	-	72.4% RRMS; 27.6% PPMS - N.A.	BMI: 25.2 ± 1.5; WC: 89.6 ± 3.6; HC: 102.7 ± 1.2; WHR: 0.87 ± 0.03	10 - 80.0	-	RRMS - 23.4 ±4.7	BMI:23.4 ± 1.2; WC: 83.8 ± 2.7 HC: 97.2 ± 2.4 WHR: 0.87 ± 0.02	D	BMI, WC, HC
			Group 2: 10 - 80.0	-		BMI: 24.2 ± 0.9; WC: 89.6 ± 2.6; HC: 102.0 ± 1.0; WHR: 0.89 ± 0.02						
			Group 3: 8 - 37.5	-		BMI: 24.9 ± 1.1; WC: 96.3 ± 2.5; HC: 105.1 ± 3.0; WHR: 0.95 ± 0.05						
25	Wens et al., 2016 [63]	RCT	15 - 60.0	42 ± 3	RRMS - 2.7 ± 0.3	%F: 37.1 ± 2.5; FFM: 41.9 ± 2.6	7 - 71.4	44 ± 2	RRMS - 2.0 ± 0.3	%F: 38.8 ± 3.2; FFM: 36.8 ± 3.2	PA	BC (DEXA)
26	Yadav et al., 2016 [64]	RCT	26 - 96.9	40.8 ± 8.86	N.A. - 2.72 ± 1.05	BMI: 29.6 ± 1.4	27 - 89.6	40.9 ± 8.48	N.A. - 2.22 ± 0.90	BMI: 28.1 ± 1.3	D	BMI
27	Wens et al., 2015 [62]	RCT	29 - 58.6	48 ± 2	RRMS: 17; CP: 17 - 3.25 ± 0.2	BMI: 22.6 ± 0.9; %F: 35.3 ± 1.5; FFM: 41.8 ± 1.7	15 - 53.3	49 ± 2	RRMS: 11; CP: 4 - 3.36 ± 0.4	BMI: 22.9 ± 1.3; %F: 36.5 ± 2.4; FFM: 41.8 ± 2.4	PA	BC (DEXA)
28	Bisht et al., 2014 [76]	Single-arm open-label intervention study	9 - 90.0	52.4 ± 4.1	SPMS - N.A.	BMI: 25.5 ± 4.7	-	-	-	-	Multimodal intervention	BMI
29	Pilutti et al., 2014 [34]	RCT	35 - 73.2	48.4 ± 9.1	N.A. - 3.5 (4.25) Self-reported	BMI: 27.9 ± 7.7; BMD: 1.10 ± 0.09 g/cm^2^; %F: 33.7 ± 8.8; LBM 48.5 ± 1.0	37 - 78.0	49.5 ± 9.2	N.A. - 3.5 (4.5) Self-reported	BMI: 27.6 ± 6.4; BMD: 1.102 ± 0.100 g/cm^2^; FM% 35.7 ± 7.8 LBM 46.4.2 ± 8.9	PA	BMI; BC, BMD (DEXA)
30	Schmidt and Wonneberger, 2014 [72]	Pre-test–post-test longitudinal design	60 - 76.7	38.3 ± 8.4	RRMS - 1.9 ± 0.6	BMI: 24.7 ± 4.2; %F:19.2 ± 3.7	-	-	-	-	PA	%F (skinfolds)
31	Carter et al., 2013 [41]	RCT	14 - 87.5	39.5 ± 6.5	N.A. - 3.0 ± 1.1	BMI:26.7 ± 5.7; WHR: 0.79 ± 0.07	12 - 82.3	40.9 ± 8.7	N.A. - 3.1 ± 1.7	BMI: 26.6 ± 5.4; WHR: 0.80 ± 0.08	PA	BMI, WC, HC
32	Learmonth et al., 2012 [58]	RCT	15 - 75.0	51.4 ± 8.06	N.A. - 6.14 ± 0.36	BMI: 28.7 ± 5	10 - 71.4	51.8 ± 8.0	N.A. - 5.82 ± 0.51	BMI: 31.4 ± 5.9	PA	BMI
33	Dalgas et al., 2010 [57]	RCT	15 - 66.7	47.7 ± 10.4	N.A. - 3.7 ± 0.9	Weight: 70.1 ± 14.2; %F BIA: 28.4 ± 6.4; %F Sk: 31.7 ± 6.8	16 - 62.5	49.1 ± 8.4	N.A. - 3.9 ± 0.9	Weight: 66.9 ± 15.2; %F BIA: 27.9 ± 9.7; %F Sk: 31.1 ± 8.0	PA	%F (skinfolds, BIA)
34	Castellano et al., 2008 [69]	Non-randomized controlled trial	11 - 72.7	40 ± 10	RRMS - (0–5.5)	BMI: 24 ± 4; %F: 35.6 ± 8	11 - 72.7	40 ± 10	Healthy	BMI: 27 ± 5; %F: 37.6 ± 9	PA	BMI, %F, WHR
35	Fragoso et al., 2008 [70]	Pre-post test longitudinal design	9 - 88.9	35.4 ± 11.6	8% RRMS; 1% SRMS - 1.8 ± 1.8	BMI: 24.4 ± 4.1 %F: 19.4 ± 6.6 FFM: 47.6 ± 9.4	-	-	-	-	PA	BMI; %F
36	White et al., 2006 [71,80]	Pre-test–post-test longitudinal design	12 - 100.0	47.3 ± 4.7	RRMS - N.A.	BMI: 25.4 ± 5.9 %F: 33.5 ± 7.2	-	-	-		PA	BMI, %F (skinfolds)

Abbreviations: BC: body composition; BIA: bioimpedance analysis; BMC: bone mineral content; BMD: bone mineral density; BMI: body mass index; CP: chronic progressive; D: dietary intervention; DEXA: dual X-ray absorptiometry; FFM: fat free mass (kg) FM: fat mass (kg); LBM: lean body mass; HC: hips circumference; MS: multiple sclerosis; PA: physical activity/exercise/rehabilitation; PPMS: primary progressive multiple sclerosis; PS: probiotic supplementation; QUS: quantitative ultrasonometry; RCT: randomized control trial; RRMS: relapsing-remitting multiple sclerosis; SPMS: secondary progressive multiple sclerosis; WC: waist circumference; WHR: waist-to-hip ratio; WHTR: waist to height ratio; %F: fat percentage; ° Median (range).

**Table 3:** Summary of studies’ characteristics.

Study design	- 22 (61.1%) randomized controlled trials (RCTs) [32,33,35,38,39,41,42,44,49,50,51,52,53,54,55,56,57,58,59,60,61,62,63,64,65,66]; - 3 (8.3%) non-RCTs [67,68,69]; - 8 (22.2%) were pre-test–post-test longitudinal trials [40,45,46,70,71,72,73,74]; - 3 (8.3%) quasi-experimental open-label studies.
Comparator	- 3 (8.3%) studies involved healthy people [67,68,69]; - 14 (38.9%) studies involved other MS patients [32,33,35,38,39,49,50,51,53,55,56,60,61,62,63,65,66]; - 19 (52.8%) studies had no comparator.
Phenotype of MS	- 14 studies (38.9%) included only RRMS patients [32,38,39,40,42,50,51,59,63,67,69,71,72,73,77]; - 12 papers (33.3%) included different typologies of MS, in particular: - SPMS [35,46,49,55,56,65,75,76,78]; - PPMS [33,55,60,65,75]; - CP [53,61,62], PRMS [46]; - all types of MS [66]. - 10 (28.6%) studies did not report this information [34,41,44,45,52,57,58,64,68,74].
EDSS scale	- 16 studies (44.4%) included patients with an EDSS score on average below 3.0 [32,39,40,42,51,53,57,59,62,63,64,66,67,68,70,72,74,77]; - 9 papers (25.0%) did not report this information in their studies [33,44,45,46,50,52,60,75,76];
Sample size	- 19 studies (52.8%) involved 15 patients or less in the intervention group [33,38,41,44,45,50,52,53,57,58,60,62,65,66,67,69,70,71,76]; - 2 studies (5.6%) involved more than 50 patients [40,72].
Gender of the intervention sample	- 30 studies (83.3%) included more than 50% of females in the intervention sample [32,33,34,35,38,40,41,42,45,46,49,51,52,53,56,57,58,60,61,62,63,64,66,67,69,70,72,73,74,75,76,77,78]; - 2 studies (5.5) included less than 50% of females in the intervention sample [55,68]; - 4 studies (11.4%) involved only females [50,59,65,71].
Age of the intervention sample	- 22 studies (61.1%) involved patients over 40 years of age on average [33,34,35,38,40,42,45,49,50,52,53,55,56,57,58,61,62,63,64,65,66,68,69,71,73,74,75,76,78]; - 2 studies (5.6%) did not specify the mean age for their samples [44,60].

**Table 4:** Physical activity and multimodal interventions.

	Study	Intervention Group					Comparator	Key Findings
		Characteristics of Intervention	Duration	Times (mins)	Freq (x/wk)	Intensity	Characteristics of Intervention	
1	Straudi et al., 2022 [55]	Task-oriented circuit training (TOCT) (2 weeks supervised and 12 weeks home-based)	14 weeks	60	3		Usual care	=BMI
2	Keytsman et al., 2021 [66]	Periodized HIIT training program	12 weeks	60	Week 1, 3: 3; Week 2: 2	Low/moderate	Classic endurance intervention	↑ BMI in IG =F% and FFM in IG ↓ %F in CG
3	Montealegre et al., 2020 [44]	Power and hypertrophic training programs	7 weeks	-	H: 65–80% RM; P: 30–70% RM	-	-	↓ BMD Hypertrophic group
4	Keytsman et al., 2019 [68]	High-intensity exercise home-based program (cycling)	6 months		3		High-intensity home-based exercise program (cycling) (same)	↓ weight and BMI in MS group =%F and FFM in both groups
5	Keytsman et al., 2019 [74]	High-intensity concurrent training (HICT)	12 weeks		5 in two weeks	-	-	=%F and LBM
6	Orban et al., 2019 [38]	High-intensity aerobic exercise program	8 weeks	30	4	70% of maximal HR	Guided static stretching program for 30 min·d—1, 4 d·wk—1, for 8 wk	↓ %F ↑ LBM
7	Pareja et al., 2019 [45]	Resistance training	10 weeks					=%F and BMD
8	Barry et al., 2018 [67]	Short-term cycle ergometer training	8 weeks	30	1	65–75% age-predicted max heart rate	Short-term cycle ergometer training (same)	↓ BMI in the MS group
9	Duff et al., 2018 [33]	Pilates and massage therapy	12 weeks	50	2		Massage therapy (1/w, 1 h)	=%F and LBM
10	Eftekhari and Etemadifar, 2018 [50]	Clinical mat Pilates	8 weeks	30–40	3	low to moderate	No Pilates	↓ BW, BMI, WC, HC, %F =WHR and FFM
11	Khademosharie et al., 2018	Resistance and endurance training program	24 weeks	Not fixed	3	gradually increased	No additional exercise program	↓ %F
12	Negaresh et al., 2019 [42]; Mokhtarzade et al., 2018 [51]	Short-term interval exercise training	8 weeks		3	60–70% peak power	No additional exercise program	=BMI
13	Yang et al., 2018 [46]	Controlled whole-body vibration training	8 weeks	5	3	-	-	↑ BMD
14	Mokhtarzade et al., 2017 [59]	Aerobic interval training	8 weeks		3		No additional exercise program	↓ weight, BMI and %F
15	Wens et al., 2016 [63]	Endurance and resistance training	24 weeks	increasing (from 45 to 75)	2.5	mild to moderate	No additional exercise program	=BMI, %F ↑ LBM
16	Wens et al., 2015 [61]; Wens et al., 2017 [53]	High-intensity training divided into the following: - High-intensity interval (HITR); - High-intensity continuous cardiovascular training (HCTR)	12 weeks	Not fixed	5 in two weeks	increasing	No additional exercise program (sedentary)	↓ %F in HITR and HCTR groups ↑ LBM HCTR group
17	Wens et al., 2015 [62]	Resistance and endurance training	24 weeks	increasing	2.5 (5/2 weeks)	increasing	No additional exercise program	↑ LBM =weight and %F
18	Bisht et al., 2014 [76]	Modified paleolithic diet with supplements, stretching, strengthening exercises with electrical stimulation of trunk and lower limb muscles, meditation, and massage	12 months				-	↓ weight and BMI
19	Pilutti et al., 2014 [34]	Internet-delivered physical activity behavioral intervention (primarily walking)	6 months		Monthly appointments with a behavioral coach		No behavioral intervention	=BMI and LBM ↓ %F ↑ BMD
20	Schmidt and Wonneberger, 2014 [72]	Individualized aerobic endurance exercise	12 months	30	3			↓ %F
21	Carter et al., 2013 [41]	Pragmatic physical intervention (range of aerobic and body conditioning exercise options)	10 weeks	60	3 (2 supervised and 1 at home)	50 to 69% age predicted max HR	No additional exercise program	=BMI and WHR
22	Dalgas et al., 2010 [57]	Lower body progressive resistance training program	12 weeks		2		No additional exercise program	=%F
23	Learmonth et al., 2012 [58]	Leisure exercise intervention (including mobility, balance, and resistance exercises)	12 weeks	60	2		No additional exercise program	=BMI
24	Castellano et al., 2008 [69]	Aerobic training program (cycle ergometry)	8 weeks	30	3	60% peak O_2_ uptake	Aerobic training program (cycle ergometry) (same)	=BMI, WHR and %F
25	Fragoso et al., 2008 [70]	Gradual stretching, resistance, and aerobic exercises adapted for each individual	20 weeks	60–90	3		No additional exercise program	=%F and FFM
26	White et al., 2006 [71,80]	Individualized lower-extremity progressive resistance training	8 weeks	30		increasing		=BMI and %F

Abbreviations: BMD: bone mineral density; BMI: body mass index; HC: hips circumference; FFM: fat free mass; LBM: lean body mass; WHR: waist-to-hip ratio; WC: waist circumference; %F: Fat percentage; ↑ significant increase; ↓ significant decrease.

**Table 5:** Dietary interventions.

	Study	Intervention Group		Comparator	Key Findings
		Characteristics of Intervention	Duration	Characteristics of Intervention	
1	Brenton et al., 2022 [77]	Ketogenic diet administration	6 months	No comparator	↓ BMI, WC, FM, FFM
2	Aristotelus et al., 2021 [32]	Dietary supplement formula, NeuroaspisTM PLP10 (omega-3, omega-6 PUFAs, specific antioxidant vitamins)	24 months	Placebo	=BMI, %F in both groups
3	de la Rubia Ortí et al., 2021 [49]; Platero et al., 2021 [78], 2020 [35]; Benlloch et al., 2020 [56]	Isocaloric Mediterranean diet plus 60 mL of coconut oil and 800 mg epigallocatechin gallate	4 months	Isocaloric Mediterranean diet plus placebo	↓ BMI, %F; ↑ FFM
4	Wingo et al., 2020 [73]	Low glycemic load diet (100 g of carbohydrate and GL of ≤45 points/1000 kcal daily	12 weeks	No comparator	↓ BMI, FM, FFM
5	Benlloch et al., 2020 [75]	Mediterranean isocaloric and ketogenic diet (adapted to each subject, 5 meals/day) 60 mL/day of coconut oil	4 months	No comparator	↓ %F, ↑ muscle mass
6	Brenton et al., 2019 [77]	Ketogenic diet administration (modified Atkins diet)	6 months	No comparator	↓ BMI, WC, FM, FFM
7	Fitzgerald et al., 2018 [52]	Group 1: daily caloric restriction: 22% daily restriction	8 weeks	Isocaloric diet	=BMI, BC, WC, HC. No significant changes among groups over time.
		Group 2: intermittent CR diet: 75% restriction, 2 days/week; 0% reduction, 5 days/week			
8	Tamtaji et al., 2017 [39]	Probiotic supplements (*Lactobacillus acidophilus*, *Lactobacillus casei*, *Bifidobacterium bifidum*, and *Lactobacillus fermentum*) on gene expression related to inflammation, insulin, and lipids	12 weeks	Placebo	=BMI in both groups
9	Yadav et al., 2016 [64]	Low-fat, plant-based diet	12 months	Usual diet	↓ BMI (more significant and faster in IG than in CG)
10	Riccio et al., 2016 [60]	Group 1 (RRTD): IFN-b and vitamin D administration + dietary prescription.	7 months	IFN-b therapy; vitamin D3 administration; no dietary or supplements prescription	=BMI, WC, HC. No significant changes among groups.
		Group 2 (RRTDI): FN-b therapy, vitamin D, dietary restriction + dietary supplements.			
		Group 3 (PPDI): PPMS patients. Cholecalciferolo administration, dietary control, supplement administration.			

Abbreviations: BMD: bone mineral density; BMI: body mass index; HC: hips circumference; FFM: fat free mass; LBM: lean body mass; WHR: waist-to-hip ratio; WC: waist circumference; %F: Fat percentage; ↑ significant increase; ↓ significant decrease.

## Supplementary Materials

The following supporting information can be downloaded at: https://www.mdpi.com/article/10.3390/jfmk8030132/s1, Figure S1: Assessment of risk of bias.
