# Supplementary material for: Effects of Exercise, Rehabilitation, and Nutritional Approaches on Body Composition and Bone Density in People with Multiple Sclerosis: A Systematic Review and Meta-Analysis
Source: J Funct Morphol Kinesiol. 2023 Sep 8;8(3):132. doi: 10.3390/jfmk8030132 (PMC10532597; doi:10.3390/jfmk8030132)
Supplement: Supplementary file 1 [file jfmk-08-00132-s001.zip › jfmk-2567961-supplementary.pdf]

Effects of Exercise, Rehabilitation, and Nutritional Approaches on Body Composition and Bone Density in People with Multiple Sclerosis: A Systematic Review and Meta-Analysis

References	D1	D2	D3	D4	D5	Overall
Straudi et al., 2022	+	+	+	+	+	+
Brenton, et al. 2022	!	+	+	!	!	!
Aristotelous et al., 2021	+	+	+	+	+	+
Keytsman et al., 2021	+	+	+	+	!	!
Benlloch et al., 2020	+	!	+	-	+	-
Montealegre et al., 2020	+	+	+	!	-	-
Wingo et al., 2020	!	+	+	+	+	!
Benlloch et al., 2019	!	+	+	-	+	-
Brenton et al., 2019	!	+	+	!	!	!
Keytsman et al. 2019a	!	+	+	+	+	!
Keytsman et al. 2019b	!	+	+	!	+	!
Orban et al. 2019	+	+	+	+	!	!
Torres Pareja et al., 2019	!	+	+	!	!	!
Barry et al., 2018	!	+	+	-	!	-
Duff et al., 2018	+	+	+	+	+	+
Eftekhari and Etemadifar et al., 2018	+	+	+	+	!	!
Khademoshaire et al. 2017	+	+	+	-	+	-
Negaresh et al., 2019	+	+	+	+	!	!
Fitzgerald et al., 2018	+	+	+	+	!	!
Yang et al., 2018	!	+	+	!	!	-
Mokhtarzade et al., 2017	+	+	+	+	!	!
Tamtaji et al., 2017	+	!	+	+	-	-
Wens et al., 2015a	+	+	+	+	+	+
Riccio et al., 2016	-	+	+	+	!	-
Wens et al., 2016	+	+	+	+	+	+
Yadav et al., 2016	+	+	+	+	+	+
Wens et sl., 2015b	+	+	+	+	-	-
Bisht et al., 2014	!	-	+	!	-	-
Pilutti et al., 2014	+	+	+	+	!	!
Schmidt and Wonneberger, 2014	!	+	+	!	!	!
Carter et al., 2013	+	+	+	+	!	!
Learmonth et al., 2011	+	+	+	+	!	!
Dalgas et al., 2010	+	+	+	+	+	+
Castellano et al., 2008	!	+	+	-	!	-
Fragoso et al., 2008	!	+	+	-	!	-
White et al., 2006	!	+	+	+	!	!

\+ Low risk
! Some concerns
\- High risk

D1 Randomisation process
D2 Deviations from the intended interventi
D3 Missing outcome data
D4 Measurement of the outcome
D5 Selection of the reported result

Figure S1. Assessment of risk of bias. [32–34,38–41,44–46,49–52,55–80].
